# Supplementary material for: Are carbon emissions trading and green financial instruments synergistic? -Comprehensive quantitative research based on content analysis
Source: PLoS One. 2024 Mar 7;19(3):e0298601. doi: 10.1371/journal.pone.0298601 (PMC10919601; doi:10.1371/journal.pone.0298601)
Supplement: S1 Data — (ZIP) [file pone.0298601.s001.zip › Data/地级市Z_F工作报告.zip!/║■▒▒/╩«╤▀╩╨/└┤╫╘╣1⁄2╓┌║┼í╛┬φ┐╦╩2╛▌═°í┐_2018.docx]

　　1、2017年工作回顾

　　过去的一年，是新一届政府开局之年。一年来，在省委省政府和市委的坚强领导下，我们以习近平新时代中国特色社会主义思想为指导，认真贯彻落实党的十九大精神，牢固树立新发展理念，坚持稳中求进总基调，积极应对经济形势复杂多变和旱涝灾害交替的双重考验，抗大灾，克大难，办大事，创大业，实现了经济社会持续健康发展。

　　——经济运行稳中有进。预计实现地区生产总值1620亿元，增长10%左右；规模以上工业增加值520亿元，增长14%左右；固定资产投资1536亿元，增长16%以上；社会消费品零售总额815亿元，增长12.5%；地方一般公共预算收入107.6亿元，可比增长11.7%；城乡居民收入分别达28732元、9365元，增长9%、10%。主要指标增速位居全省前列，稳的基础持续巩固，进的步伐不断加快，好的态势更加凸显。

　　——抗灾救灾有力有为。沉着应对有气象记录以来最长时间持续强降雨和旱涝急转带来的严重自然灾害，成功避让地质灾害26起，安全转移受灾群众2.6万人次、救助40余万人次。抢修国省干线2226处，修复水利设施1945处，修缮房屋2.34万间，恢复供水16.8万人，实现了“有饭吃、有衣穿、有水喝、有住处、有病能医”目标，农业生产保持稳定，大灾之年无大难，抗灾救灾取得全面胜利。

　　——发展气场不断集聚。成功举办了第四届国际道教论坛、第九届海峡两岸中华武术论坛等重大活动，提升了十堰知名度、美誉度和武当山国际影响力。全力冲刺第五届全国文明城市，城市面貌焕然一新，文明程度显著提升，荣获 “全国未成年人思想道德建设工作先进城市”。积极参与央视魅力中国城竞演，获评“最受观众喜爱魅力城市”，展示了奉献坚韧、开放包容、创新进取的十堰风采。十巫高速、十淅高速开工建设，汉十高铁、地下综合管廊等一批重大基础设施顺利推进，东风易捷特新能源乘用车、双星东风轮胎4.0新工厂等一批项目签约落户。荣获“全国美丽山水城市”，老工业基地调整改造和产业转型升级受到国务院表彰。

　　一年来，我们主要做了以下工作：

　　（一）认真学习贯彻落实党的十九大精神。

　　（二）加快转型升级，提升发展质效。

　　（三）狠抓项目投资，夯实发展基础。

　　（四）坚持全域统筹，促进协调发展。

　　（五）深化改革开放，增强发展活力。

　　（六）修复生态环境，推进绿色发展。

　　（七）强力脱贫攻坚，增进民生福祉。

　　（八）加强社会治理，促进和谐稳定。

　　与此同时，全市外事侨务、气象、档案、地震、人防、残联、老龄、地方志等各方面工作都取得了新的成绩。

　　2、2018年工作安排

　　今年是全面贯彻党的十九大精神的开局之年，也是我市推进脱贫攻坚、决胜全面建成小康社会的关键一年，做好今年的政府工作意义十分重大。

　　今年政府工作的总体要求是：全面贯彻党的十九大精神，以习近平新时代中国特色社会主义思想为指导，坚持稳中求进工作总基调，坚持新发展理念，牢牢把握高质量发展的根本要求，统筹推进“五位一体”总体布局和协调推进“四个全面”战略布局，以供给侧结构性改革为主线，围绕市委提出的打造区域性增长极、建设社会主义现代化强市目标，坚持干在实处、走在前列，以项目建设为抓手，大力推进稳增长、促改革、调结构、强生态、惠民生、防风险各项工作，打好精准脱贫、转型升级、污染防治攻坚战，促进经济社会持续健康发展，以优异的成绩迎接建市50周年。

　　今年发展的主要预期目标是：地区生产总值增长8%左右，力争在实际工作中取得更好成果；规模以上工业增加值增长8%以上；固定资产投资增长14%；社会消费品零售总额增长11%；地方一般公共预算收入增长8.5%；外贸出口高于省定任务；城镇常住居民、农村居民人均可支配收入分别增长8%、9%；居民消费价格指数和节能减排控制在省定目标以内。

　　围绕上述目标任务，重点做好八个方面工作：

　　（一）突出乡村振兴，坚决打好脱贫攻坚战

　　坚持农业农村优先发展，大力实施乡村振兴战略，集中优势资源，向深度贫困地区聚焦发力，整体提升脱贫质量，确保同步进入小康。

　　——全力攻克深度贫困堡垒。坚持精准扶贫、精准脱贫，把提高脱贫质量放在首位，注重激发贫困人口内生动力，巩固提升扶贫成果。突出“六个精准”，切实把脱贫举措落实到村到户到人。推进产业扶贫，因村因户发展特色产业，实现产业项目应扶尽扶。推进易地扶贫搬迁，实现10.7万贫困人口“搬穷窝、换穷业”，全面完成易地扶贫搬迁任务。统筹推进科技、教育、卫生、文化、社保、金融等综合扶贫。集中力量攻克深度贫困，切实落实“三个新增”政策。实施贫困村公路硬化扩宽、安全饮水、电网改造、通信网络等提升工程，改善基础设施条件。推进“四双”驻村帮扶，有效提升“三率一度”。开展扶贫领域腐败和作风问题专项治理，严把“精准识别、精准退出”两大关口，严防“被脱贫”“数字脱贫”。确保14.5万人脱贫，134个村出列，丹江口市、张湾区、茅箭区和武当山特区脱贫“摘帽”。

　　——加快建设现代农业。深化农业供给侧结构性改革，走质量兴农之路，加快构建现代农业产业体系、生产体系、经营体系。做大基地规模，提升产业素质。深入实施“61”产业强农计划，新增标准化基地10万亩以上，改造老基地15万亩。开展万亩专业乡镇创建，重点建设竹房百公里茶叶长廊和汉江百公里林果长廊，争创国家级“特优区”。新建高标准农田11万亩、补建19万亩，确保粮食总产稳定在10亿公斤以上。做强龙头企业，提升品牌效益。实施龙头企业培育提升工程，大力发展新型经营主体，新增规上农产品加工企业10家、过亿元企业5家、农民专业合作社200家以上。大力实施“一县一品”、“一村一品”，加大郧巴黄牛、马头山羊、武当道茶等系列产品开发力度，推进丹江口库区生态渔业转型，新增“三品一标”品牌10个以上。做优特色园区，延伸产业链条。主攻农产品加工短板，支持5个省级现代农业产业园区建设，力争农产品加工产值突破600亿元。

　　——积极推进乡村振兴战略。按照产业兴旺、生态宜居、乡风文明、治理有效、生活富裕的总要求，遵循乡村发展规律，坚持城乡融合发展，因地制宜扎实推进乡村振兴战略。深入推进农村土地、集体产权制度改革，加快农村产权交易市场体系建设。探索推进“三权分置”多种实现形式，创新完善农业支持保护制度。扎实推进美丽乡村建设，以“厕所革命”为牵引，抓好环境建设与整治，加快补齐农村公共服务、基础设施、信息流通等方面短板。认真做好丹江口水库移民安置总体验收国家终检迎检工作。加强“三农”工作队伍建设，大力实施“三乡工程”。推进“十星”创建等农村精神文明建设，依托村规民约，培育农村新风尚。加强农村基层基础工作，健全自治、法治、德治相结合的乡村治理体系。

　　（二）突出转型升级，加快建设现代汽车城

　　坚持以提高质量和效益为中心，推动质量变革、效率变革、动力变革，加快构建“一主四大四新”现代产业体系。

　　——改造提升汽车产业。以绿色化、智能化、轻量化、网联化发展为主线，推动汽车与新一代信息技术、新能源、新材料等新兴产业深度融合。做强做大东风十堰基地，全力服务东风汽车集团有限公司。全面落实十堰与东风政企共建50周年深化战略合作协议，加快推进东风易捷特新能源乘用车、东风M9T轻型发动机、东风商用车总装及车架业务优化等重点项目建设。加快发展新能源汽车，推进整车专用化。主攻“专精特新轻”，巩固专用车优势地位，扩大新能源汽车生产规模，推进驰田金刚10万辆专用车等项目建设。实施零部件提升工程，整体提高配套服务能力。服务推进好东风装备、动力总成、零部件、双星东风轮胎4.0新工厂等园区建设。滚动实施“千企千亿”技改工程，重点突破汽车电子系统、电机驱动系统等核心技术，推进传统制造业向先进制造业裂变升级。确保新增规上企业50家，生产各类汽车68万辆，完成规上工业总产值2300亿元以上。

　　——调整优化工业布局。坚持扩总量、提质量、优结构、增效益并重，整体优化完善工业布局。加大“退二进三”、异地置换力度，加快新型工业化示范基地建设。大力发展高端装备、智能制造、新能源、新材料等战略性新兴产业，培植壮大生物医药、食品饮料等6个省级重点成长型产业集群，重点推进锂诺新能源电池、国药集团、长江医药等一批项目。积极争取老工业区搬迁改造纳入国家示范工程试点，支持东风十堰基地主辅分离。以十堰经济技术开发区为龙头，推进白浪工业园、六里坪工业园、东城经济开发区融合发展，启动新经济产业园建设，构建“一区三园”发展格局。整合十堰生态滨江新区、十堰工业新区，申报创建国家级高新区。

　　——强化创新驱动。深入推进 “中国制造2025”、“互联网+”行动计划，加快新旧动能转换，促进传统动能焕发生机、新动能快速壮大。大力实施新兴产业培育和传统产业转型升级行动计划。加快创新创业平台建设，鼓励创建一批市级以上重点实验室、工程技术研究中心。大力推进中小企业成长工程，加快建设一批众创空间、“双创”示范基地。推进科技成果转化应用，实施一批重点科技专项。力争新增高新技术企业20家，高新技术产业增加值230亿元以上。培养造就一批科技人才和高水平创新团队，弘扬企业家精神和“工匠精神”，大力实施品牌强市、质量强市和标准化战略，开展质量提升、品牌创建行动，积极争创 “全国质量强市示范城市”。

　　（三）突出全域旅游，大力发展现代服务业

　　加快做强做大生态文化旅游业，突破性发展商贸物流业，创新发展新兴服务业，坚定不移把服务业打造成新的支柱产业。

　　——培育壮大旅游业。围绕打造千亿级支柱产业，实施“两区三带”全域旅游发展规划，着力建设“东方国际休闲养生旅游目的地”。科学规划、整体推进，加快“全域风景区、全域生态区、全域水源区”建设，整体改造提升大武当、十堰城区、汉水-堵河等重点区域；突出重点、提升品质，加大旅游基础设施建设力度，加快补齐旅游产品线路单一、市场营销力度不够、附加值不高等短板，着力构建“山水城互动、文旅农互融、景点景区互联、各要素配套”格局；全域统筹、差异发展，以武当山为龙头，以城区和各县域为支撑，精心打造“武当369”等特色旅游品牌。实施一批旅游重点工程，加快推进神农峡岩屋沟景区、赛武当云龙谷、子胥湖生态新区、上津古镇等项目建设。推进3家4A级景区创建，提档升级5家3A级景区，力争接待游客6300万人次，实现旅游收入570亿元。

　　——提档升级商贸流通业。加快消费升级，推动“老字号”品牌发展，改造提升传统服务业，扩大线上线下互动消费。支持骨干流通企业建设跨区域配送中心，推进批发出城、零售进城，合理布局城区零售网点，建设社区便民示范店30家。开工建设名创吾悦广场、群利物流园，加快推进林安国际、佰昌商贸物流园、昌升国际商贸城、华西农商城等在建项目，新增A级物流企业3家以上、省重点物流企业3家以上。

　　——加快发展现代服务业。围绕完善生产要素市场、打造资源要素集聚区，加快构建生活性服务业繁荣丰富、生产性服务业支撑强劲、公共服务保障有力的现代服务产业体系。重点培育壮大健康养老、现代金融、文化创意、节能环保、信息服务、科技服务等新业态。以汽车汽配商品交易会等会展活动为载体，打造国家级汽车展销平台。加快发展农村电商，争取实现电商进农村全覆盖，支持丹江口市、房县、郧西县、郧阳区、张湾区创建全国电子商务进农村综合示范县，支持竹溪争创全国电商百强县。力争服务业增加值650亿元、增长9%以上。

　　（四）突出项目投资，强力打造增长引擎

　　今天的投资结构决定明天的产业结构，必须充分发挥投资“定海神针”作用，为长远发展和转型升级谋势蓄力。

　　——大视野谋划项目。千方百计扩大有效投资，密切跟踪国家产业投资政策，深度挖掘我市资源优势，找准结合点，精准有效对接，方案化、工程化谋划项目。围绕老工业区搬迁改造、生态环境修复、地质灾害治理、移民后扶、保障性安居工程等重点领域，积极向上争取财政预算投资。加大PPP项目推介力度。加强重大项目“两库”建设，力争储备亿元以上重大项目1000个、开工建设亿元以上重大项目400个以上。

　　——优服务引进项目。进一步强化招商引资“首位度、精准度、多维度”，建立“一主四大四新”产业发展与精准招商联动机制。加快健全完善招商引资政策体系，更大力度推进优势资源招商、产业链招商、以商招商、专业招商、专班招商。加强项目跟进与企业对接，积极参加鄂京、鄂沪等主题招商活动。重点抓好先进制造业、农产品加工、文化旅游等产业招商，确保新引进注册规模项目150个、近三年引进项目投产110个以上。

　　——结硬账落实项目。继续坚持重大项目首席服务官、重大项目工作专班、项目拉练等有效做法，强化督办落实，确保完成年度投资计划。加快推进汉十高铁、京能热电、夹河关水电站、孤山航电枢纽、十堰高铁站、火车站北广场、武当山机场改扩建、郧阳汽车小镇等重点项目建设，确保京能热电首台机组投产，热力管网建设全面完工，十竹长输管线顺利通气。全力抓好十巫高速十堰段、十淅高速十堰段、汉十高速武当山互通、十堰生态滨江新区至武当山一级路等重大基础设施建设，推进十西高铁开工建设。

　　（五）突出改革开放，切实增强群众获得感

　　始终坚定不移深化改革、扩大开放，向改革要动力，向开放要活力，全面拓展改革的深度和开放的广度。

　　——继续深化供给侧结构性改革。突出抓重点、补短板、强弱项，持续推进“三去一降一补”。认真落实供给侧结构性改革方案，实施去库存、降成本等七个专项行动。巩固去产能成效，严格执行环保、能耗、用地、质量、安全等标准，有效处置“僵尸企业”。用足用活棚改政策，加大棚户区改造力度，积极培育发展住房租赁市场，加快建立多主体供给、多渠道保障、租购并举的住房制度，让广大群众住有所居。加大金融支持服务实体经济力度，防范化解风险，用好用活信贷杠杆，优化金融生态环境。清理规范政府性基金及中介服务事项，有效降低企业成本。

　　——持续推进关键领域改革。深化 “放管服”改革，推进相对集中行政审批许可权改革，构建“一窗受理、一表申报、一章审批、一次办结”新模式和审管联动新机制，实行政务服务“一网覆盖、一网通办”，促进大众创业、万众创新。整合资源建设十堰大数据中心，完善政务服务数据库，优化升级智慧政务平台。深化“多证合一、一照一码”商事登记制度改革，实行“同城通办”服务。深化财税体制改革，设立武当产业基金，落实“营改增”等减税减负政策。严格预算执行，全面实行绩效管理。加大财政资金统筹力度，提高资金使用效益。完善各类国有资产管理体制，深入推进国有企业改革，促进国有资产保值增值。加强资本市场建设，扩大直接融资，推进企业上市与债券发行。巩固公共资源交易平台整合成果，建立公开、公平、公正的交易环境。加快“信用十堰”建设，加强信息共享、协同监管和联合惩戒，落实信用修复机制。继续深化户籍管理制度改革。完善国土、统计等改革，做好第三次土地调查和第四次全国经济普查。实行更加积极开放有效的人才政策，为人才干事创业创造良好环境。深化事业单位改革，强化公益属性，推进政事分开、事企分开、管办分离。统筹推进社会民生领域改革，不断满足群众美好生活需要。

　　——全面提高开放水平。强化开放举措，创优营商环境，着力打造内陆开放前沿。深度融入“一带一路”建设，积极对接湖北自贸区，大力发展开放型经济。优化开放布局，加大开放力度，放宽市场准入，支持民营经济发展，大力引进各类市场主体。用好用足外贸出口政策，促进外贸扩市场、增份额。推广国际贸易“单一窗口”，扩大汽车及零部件、绿色有机食品等优势产品出口规模。深层次广领域开展与北京等地对口协作，扎实推进合作项目建设。积极开展丹江口库区及上游地区和秦巴山片区经济协作，扎实推进援疆工作。

　　（六）突出区域协调，推进城乡融合发展

　　充分发挥我市山水、人文、生态、产业优势，深入实施区域协调发展战略，促进城乡一体、城乡和谐。

　　——优化提升城市品质。高标准规划建设管理，完善市政设施，提升城市功能，努力让城市生活更方便、更舒心、更美好。加快推进城市总规修编报审并实施，系统推进专规详规编制，完成“退二进三”规划编制，统筹推进“多规合一”。继续推进管廊城市、海绵城市等项目建设。启动实施百二河生态修复工程，着力打造市民“15分钟生活休闲圈”。启动北京路改造、天津路大修等一批重点道路建设，加快推进三峡路、火箭路、武当路复线、林荫大道等续建工程，完成重庆路、浙江路等改造工程。提高城市科学化管理水平，更大力度解决城市交通拥堵、出行不畅、购物不便、市政设施落后、公共服务滞后等“城市病”，打通一批微循环道路，推进老旧小区综合改造、加装电梯，解决好老百姓关心的“天大的小事”。加大住房保障力度，完善房地产市场平稳健康发展长效机制，完成1.26万户棚改任务。加快建设生态滨江新区，推进汉江四桥及连接线等项目建设，加快“山城”向“山水城”迈进步伐。

　　——促进县域全面发展。坚持“四化同步”，全面提升县域发展水平。推进城区与各县市协调发展，突出县域优势，彰显县域特色，健全完善充满生机与活力的县域发展机制。鼓励各县市区争先进位、比拼赶超、竞相发展，提升支点实力。推进产城融合，强化产业支撑，做强做大县域经济。用好用足县域调度资金、产业发展基金，打造一批百亿级产业集群。支持丹江口市食品饮料、郧阳区香菇生产加工、房县黄酒发酵食品、郧西县板材加工、竹山县绿松石、竹溪县绿色有机农产品等产业集群发展壮大。力争县域工业增加值增长9%以上。

　　——扎实推进两带建设。深入推进以人为本的新型城镇化，加快城际快速交通体系建设，增强城市辐射带动功能，促进形成中小城镇协调发展格局。纵深推进竹房城镇带、汉江生态经济带建设，新建高标准基地3万亩以上，建设9个农村新社区示范片区、10个规模化产业示范基地。深入开展特色小镇和人居环境示范村创建，推进竹溪县汇湾贡茶小镇等11个国家级重点镇、8个全省重点中心镇、6个全省特色镇建设，改造农村危房1万户以上。

　　（七）突出绿色发展，全力建设美丽十堰

　　坚持节约优先、保护优先、自然恢复为主，建设天蓝、地绿、水清的美丽十堰，还自然以宁静、和谐、美丽。

　　——持续整治环境污染。坚持全民共治、源头防治，坚决打好大气、水、土壤污染防治攻坚战。持续改善环境质量，巩固城区空气质量达标成果，全市地表水水质优良比例87.5%以上，城市集中式饮用水源地水质达标率100%。全面完成神定河水质净化、泗河污水处理扩能改造等工程，新建改造污水管网40公里，确保85座乡镇污水处理厂竣工运营。完善“河长制”长效管理，确保“五河”等河流水质达到考核标准，丹江口水库水质稳定达到Ⅱ类以上。全面实施“土十条”，加强土壤污染管控和修复。加大节能减排力度，完成单位GDP能耗、二氧化碳排放控制性目标任务。狠抓突出环境问题整改，坚决按期完成整改任务。

　　——加大生态系统保护与修复力度。把修复长江生态环境摆在压倒性位置，共抓大保护、不搞大开发。严格落实生态红线管控，启动勘界定标工作，进一步明确管控范围内的正面清单和负面清单。持续开展“绿盾”“精准灭荒”等行动，实施生态系统保护和修复重点工程，优化生态安全屏障体系。深化国土资源节约集约示范市创建。守牢耕地红线，改良土壤、提升地力，确保耕地保有量303万亩以上，基本农田245万亩以上。做大绿色增量，护好绿色存量，提升绿色质量，实施好天保、长防、新一轮退耕还林等工程，新造林12万亩、义务植树1000万株以上。加大地质灾害防治力度，持续推进石漠化防治、水土保持、小流域综合治理等生态项目建设。

　　——努力创建全国生态文明建设示范市。增强绿色发展意识，提高绿色发展能力，让老百姓享受更多的绿色发展成果。加快发展节能环保、清洁能源等产业，建立健全绿色低碳循环发展经济体系。制订实施生态文明建设示范市三年行动方案，全面开展领导干部自然资源资产离任审计、自然资源资产负债表试点等工作，完善失职追责考评机制。

　　（八）突出民生为本，不断满足群众美好生活需要

　　坚持以人民为中心，倾注更大力量增进民生福祉，使群众获得感、幸福感、安全感更加充实、更有保障、更可持续。

　　——大力保障和改善民生。优先发展教育事业，办好人民满意的教育。推进普惠性公益幼儿园建设，巩固义务教育均衡发展成果，推进普及高中阶段教育，打造职业教育特色品牌，促进高等教育内涵式发展。抓好柳林中学、车城高中续建，市一中学生宿舍楼新建等项目建设；引入社会资本多渠道办学，缓解城区学位紧张矛盾。坚持就业优先战略和积极就业政策，促进更高质量和更充分就业。多渠道开发就业岗位，新增就业3.6万人，再就业1.8万人，城镇登记失业率控制在4.5%以内；推进“健康十堰”建设，为群众提供全方位全周期健康服务。持续深化医药卫生体制改革，全面取消以药养医，推进现代医院、分级诊疗、药品供应保障、综合监管等制度改革，加强基层卫生服务体系建设。抓好市太和医院太极湖医院、市中医医院业务综合楼、市妇幼保健院新院区等项目建设。实施全面“两孩政策”，持续加强出生人口性别比综合治理。

　　——健全完善社会保障体系。实施全民参保计划，巩固社会保险全覆盖成果。严格落实国家社会保险费率、待遇调整政策，稳步提高社会保险待遇。实施统一的城乡居民基本医疗保险制度和大病保险制度，完善失业、工伤保险制度。全面落实社会救助、社会福利、优抚安置等政策，健全农村留守儿童和妇女、老年人、残疾人关爱服务体系。加快推进市殡仪服务中心、市老年人养护中心等重点项目建设。

　　——建设文化强市。坚持先进文化引领，繁荣发展文化事业，深度挖掘地方特色文化资源，发展壮大文化产业，合力打造十堰文化品牌。加强文化小康建设，完善公共文化服务体系，实施文化惠民工程，不断丰富群众性文化活动。精心备战第三届湖北艺术节。加强文化遗产保护和利用，启动汽车工业文化遗产保护与利用工作，推进遇真宫复建、玉虚宫父母殿等一批文物保护项目，抓好非遗项目申报和管理。广泛开展全民健身活动，普及推广武当武术，组织参加省十五届运动会。推进国家南水北调博物馆、奥林匹克体育公园、广电传媒中心、各县市文化馆、图书馆、体育场馆等项目建设。

　　——持续深入开展全国文明城市创建活动。制定实施全国文明城市三年规划及年度方案，启动第六届全国文明城市创建工作。以文明城市创建为统领，广泛发动群众，形成创文合力，严格对标对表，建立健全机制，推进常态化、长效化创建，持续建设市容环境更美、文明程度更高的十堰。巩固国家森林城市、卫生城市创建成果，更大力度推进 “五城联创”。深入开展理想信念、社会主义核心价值观、中国梦等宣传教育，建好 “十堰好人·楷模”榜，讲好十堰故事。

　　——加强和创新社会治理。打造共建共治共享的社会治理格局，提高社会化、法治化、智能化、专业化治理水平。推进“法治十堰”建设，深入开展“七五”普法。健全预防化解社会矛盾机制，大力推进信访法治化建设。深化“平安十堰”建设，完善社会治安防控体系，积极主动预防化解社会风险，依法打击和惩治各类违法犯罪活动，保护群众人身权、财产权、人格权。牢固树立“生命至上、安全第一”理念，严格落实安全生产责任制，坚决遏制重特大安全事故发生，提升防灾减灾救灾能力。实行最严谨的标准、最严格的监管、最严厉的处罚、最严肃的问责，保障群众食品用药安全。支持国防建设和军队改革，加强全民国防教育，巩固军政军民团结，推动军民深度融合发展。

　　3、加强自身建设，提高政府执行力

　　进入新时代，对政府工作提出新的更高要求。我们必须以党的十九大精神为指引，始终不忘初心、牢记使命，努力建设人民满意政府。

　　——牢记使命担当。始终把对党忠诚、为民造福作为根本担当，牢固树立 “四个意识”，坚定 “四个自信”。坚决维护党中央权威和集中统一领导，与以习近平同志为核心的党中央保持高度一致，坚持以习近平新时代中国特色社会主义思想指导一切工作。扎实开展 “不忘初心、牢记使命”主题教育，带头贯彻执行中央和省、市委各项决策部署。始终保持昂扬状态，不断强化进取精神，勇于担当、主动作为、善干善成，在改革发展大潮中争先进、创特色。

　　——坚持实干实为。坚决把省委省政府和市委的决策部署落地落实，全面推行工作任务目标化、具体化、项目化。健全完善工作台账、督办推进、跟踪问效等机制，做到事事有时间表、人人有责任链、步步有路线图。注重培养专业能力、专业精神，提高抓落实的本领。以真抓的实劲、敢抓的狠劲、善抓的巧劲、常抓的韧劲，抓铁有痕、踏石留印抓落实。强化 “严、细、深、实”工作作风，扑下身子，真抓实干，认准的事情一抓到底，承诺的事情坚决兑现，以实干取信于民，以实绩造福于民。

　　——推进依法行政。自觉运用法治思维和法治方式深化改革、推动发展、化解矛盾、维护稳定。严格按照法定权限和程序履行职责，推进重大事项合法性审查机制全覆盖。加强公务人员法治培训，强化尊法学法守法用法意识，严格规范公正文明执法，绝不允许以言代法、以权压法、逐利违法、徇私枉法。依法接受人大法律监督、工作监督，自觉接受政协民主监督，主动接受社会和舆论监督，认真办理人大代表建议和政协委员提案，广泛听取民主党派、工商联、无党派人士意见。深入推进政务公开，促进政府行为更加科学规范、阳光透明。

　　——坚守清正廉洁。严格落实政府党组全面从严治党主体责任和 “一岗双责”，认真践行 “三严三实”，持续贯彻落实中央八项规定实施细则精神和省、市实施办法，持之以恒整治 “四风”。从严教育，从严管理，打造一支作风过硬、勤勉清正的公务员队伍。完善财政资金支出、工程项目招投标、公共资源交易等制度建设，强化关键环节监督，扎紧扎牢制度笼子。加强政务督查和效能监察，完善政绩考核评价体系，加大追责问责力度，严惩懒政怠政、失职渎职行为。支持市县监察体制改革。坚决查处违法违纪案件，以 “零容忍”态度惩治腐败。大力倡俭治奢，压缩一般性支出，做到 “三公”经费只减不增，切实把有限的财力用于发展经济、保障民生。

　　时代赋予重任，奋斗成就梦想！让我们紧密团结在以习近平同志为核心的党中央周围，在省委、省政府和市委的坚强领导下，凝心聚力，锐意进取，只争朝夕，埋头苦干，为决胜全面建成小康社会、奋力打造区域性增长极、全面建设社会主义现代化强市而努力奋斗！
